# The injury mechanism correlation between MRI and video-analysis in professional football players with an acute ACL knee injury reveals consistent bone bruise patterns

**DOI:** 10.1007/s00167-022-07002-6

**Published:** 2022-06-13

**Authors:** Pieter D’Hooghe, Alberto Grassi, Francesco Della Villa, Khalid Alkhelaifi, Emmanouil Papakostas, Raouf Rekik, Theodorakys Marin, Filippo Tosarelli, Stefano Zaffagnini

**Affiliations:** 1 Aspetar Hospital, Aspetar Orthopaedic and Sports Medicine Hospital, Sportscity Street 1, Aspire Zone, P.O. Box 29222, Doha, Qatar; 2 IIa Clinica Ortopedica e Traumatologica, IRCCS Istituto Ortopedico Rizzoli, Bologna, Italy; 3 Education and Research Department, Isokinetic Group, Bologna, FIFA Medical Centre of Excellence, Bologna, Italy

**Keywords:** ACL, Soccer, Football, Bone bruise, MRI, Pivot-shift, Video-analysis

## Abstract

**Purpose:**

To analyze the MRI features, in particular bone bruises pattern, of Anterior Cruciate Ligament (ACL) injured footballers, and to correlate them with the characteristics of injury mechanism and situation obtained from direct video footage.

**Methods:**

Nineteen professional football (soccer) players that sustained ACL injury while playing during an official match of First League Championship were included in the study. The video of injury was obtained from the Television broadcast. Knee Magnetic Resonance (MRI) was obtained within 7 days from the injury. BB and meniscal lesions were analyzed on MRI, while a video-analysis of mechanisms of ACL injury and injury dynamic were assessed from the videos.

**Results:**

The most commonly involved Bone Bruise areas in the knee were the Posterior Lateral Tibial Plateau (LTp) in 16 cases (84%) and the Central Lateral Femoral Condyle (LFc) in 11 cases (58%). Three patients (16%) had bone bruise in the Posterior Medial Tibial Plateau (MTp) while none (0%) had bone bruise in the Medial Femoral Condyle. Based on the bone bruise pattern, 11 (58%) had simultaneous LFc and LTp and were defined “Typical” while 8 (42%) had other locations or no bone bruise and were defined “Atypical”. 9 out of 11 injuries (82%) of athletes with “Typical” pattern occurred with a “Pivoting” action”, in contrast to only 1 case (12%) in those with “Atypical” bone bruise pattern (*p* = 0.0055).

The most common situational mechanism pattern on video analysis was “pressing” (*n* = 7) accounting for the 47% of the “indirect” ACL injuries. In terms of movement pattern, ten injuries (52%) occurred during a “Pivoting” movement (7 pressing, 1 dribbling, 1 tackled, 1 goalkeeping), whereas the remaining were classified as “Planting” in four cases, “Direct Blow” in four cases and “Landing”.

**Conclusion:**

A well-defined and consistent bone bruise pattern involving the posterior tibial plateau and central femoral condyle of lateral compartment is present in footballers that sustained non-contact and indirect ACL injuries during pivoting with sudden change of direction/deceleration, while heterogeneous patterns were present in those with direct contact or injury mechanisms involving high horizontal velocity.

**Level of evidence:**

Level IV.

## Introduction

The mechanisms of Anterior Cruciate Ligament (ACL) rupture represent a debated topic in nowadays sports medicine [5] due to possible advances in prevention and surgical treatment implications. In particular, understanding the movement pattern responsible of ACL failure could help in identifying individuals who are at high risk for re-injury or to [2] tailor a personalized surgical approach.

In this context, video-analysis of injuries in athletes performing popular sports with high media coverage has gained popularity in having insights of ACL injury mechanisms [3, 5, 7, 12, 14]. However, considering the public sources of videos in studies with this design, an important drawback is the lack of precise clinical and imaging information.

Another aspect which is gaining the attention of the panorama of ACL research is the pattern of bone bruises which are usually present in MRI of ACL-injured patients [1, 11, 17, 22, 23, 27]. Specifically, bone bruises are defined as areas of increased signal on short-tau inversion recovery (STIR) or T2-weighted MRI images, representing areas of increased water content due to trabecular microfractures. These contusions are therefore believed be the result of impact between the femur and tibia near the time of ACL injury, thus representing “hints” or “footprints” of a tibio-femoral contusion occurred during the ACL rupture mechanism. Moreover, their assessment has been considered providing a valuable insight into knee position near the time of ACL rupture [11, 17]. However, the injury mechanism was mainly based on patient recall or clinician assessment, while the exact injury mechanism was not investigated due to the lack of direct documentation like video footage, which are generally not available for most patients.

Due to the aforementioned current limitations of video-analysis and radiological assessment of ACL injuries, it is of interest to correlate the MRI features of ACL injuries with the exact injury mechanism obtained from video-analysis. Such investigation, which has never been performed up to now, could provide relevant insights in the understanding ACL injury mechanism and in the genesis of intra-articular lesions such as bone bruises. Considering the wide media exposure of football (soccer) and the relatively high incidence of ACL injury in this sport, footballers could represent an ideal population to be investigated. Additionally, a better understanding of the different bone bruise patterns can assist the clinician chose the appropriate treatment and prevention strategies on the short and long term as well.

Therefore, the aim of the present study was to analyze the MRI features, in particular bone bruises pattern, of ACL-injured footballers, and to correlate them with the characteristics of injury mechanism and situation obtained from direct video footage. The hypothesis was that different MRI features could be identified according to the injury mechanism.

## Material and methods

IRB approval for this study was received on August 19, 2021 by the Aspire Zone Foundation (AZF) Institutional Review Board (E202104022).

All the ACL injuries in Professional Qatar First League football players that occurred between January 2014 and January 2018 were screened. Only injuries that occurred in official matches were included in the study, and the video footage of the match was obtained. According to match reports, the exact timing of ACL injury was identified and analyzed. Since all patients were treated by the Senior Author (PdH) at a single Institution (Aspetar Orthopaedic and SportsMedicine Hospital), the surgical details and pre-operative MRIs were obtained. The personal case series of professional footballers operated by the other Senior Author (PdH) was screened as well, with the same modalities. At time of surgery, the lesion of ACL was confirmed and determined if complete or partial. Moreover, meniscal integrity was assessed; in the case of injury, lesions of medial and lateral meniscus were categorized as Anterior Horn (AH), Posterior Horn (PH), Anterior Root (AR), Posterior Root (PR), Body and Ramp. Cartilage lesions and other ligaments injuries were assessed as well.

Video-analysis of ACL injuries followed a stepwise methodology described in detail in previous papers [5, 11, 14]. Briefly, match videos were downloaded to a personal computer and cut approximately 12–15 s prior and 3–5 s after the suspected ACL injury. The videos were processed with the software Kinovea (Version 0.8.15; www.kinovea.org), allowing for accurate frame-by-frame navigation. Each injury video was reviewed by three authors (PdH, AG, FdV) to define (I) the injury mechanism, (II) the situational pattern, (III) biomechanics (kinematics) [5] and (IV) the movement pattern related to ACL injury. Each video was reviewed independently, then the reviewers met for a one-day comprehensive meeting in which agreement was found for all the items.

Injury mechanism was defined as noncontact, indirect contact or direct contact, following previously described methodology [5]. For specific analysis, noncontact and indirect contact injuries were then grouped as referred as “Indirect”. The situational pattern for “Indirect” injuries was described also according to a previously used methodology. Shortly, the situational pattern refers to the playing phase, the action and the interaction of the player with the environment [5].

Biomechanical analysis at initial contact and injury frame was carried out also when sufficient quality frontal and/or lateral plane videos were available.

Finally, the specific movement pattern insights at the suspected injury frame were identified. This item was defined based on the athlete movement at the time of injury. The “Pivoting” was defined when the player performed a sudden change of direction and deceleration, independently from ball possession. “Planting” was defined when a player gets injured planting the foot, with a vertical velocity component, but without a clear change of direction or whole-body rotation, whereas “Landing” refers to a landing from a high jump. Direct contact injuries were referred for this item as “Direct Blow”.

### MRI evaluation

**Fig. 1:**
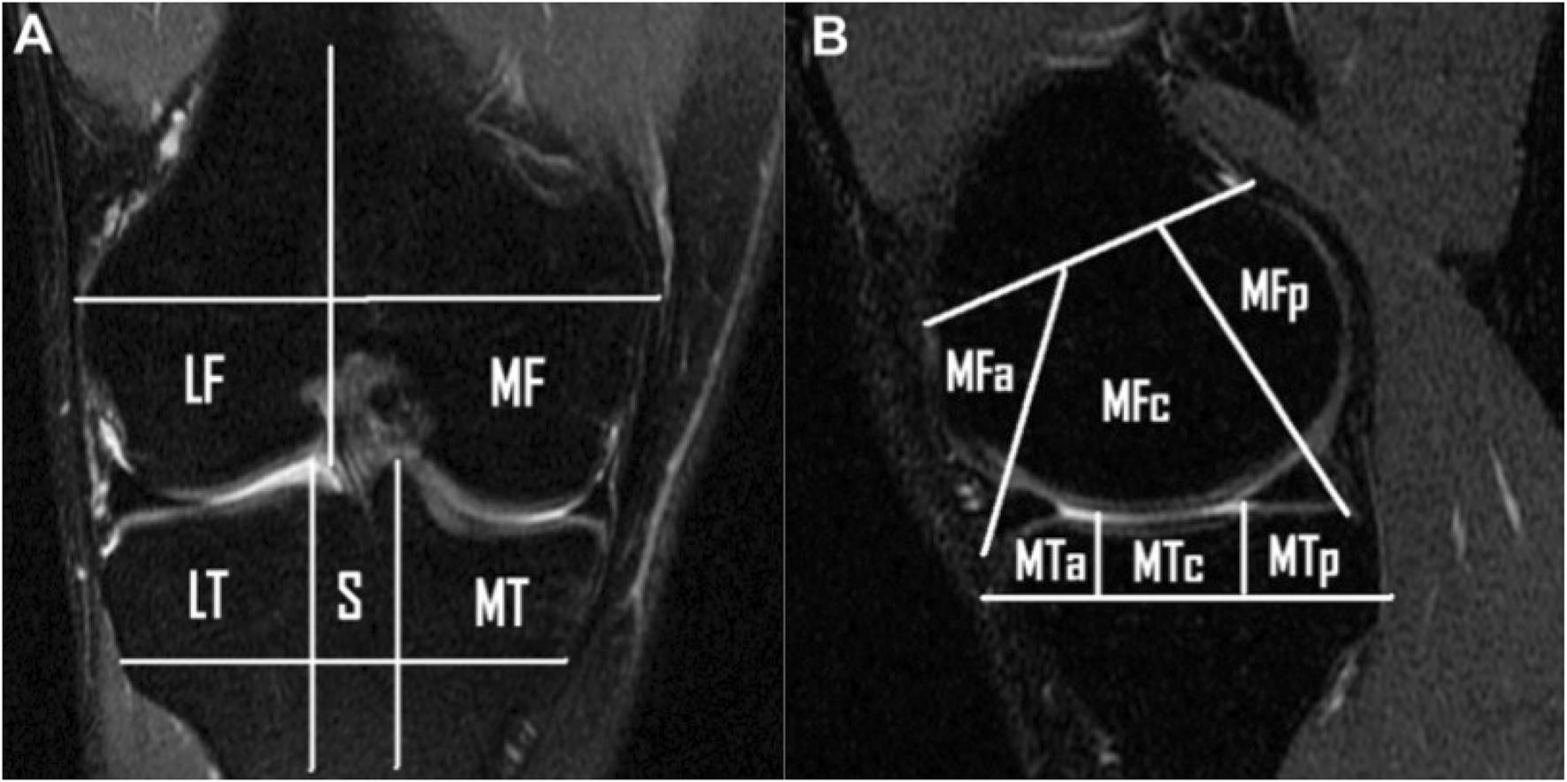
Sub-division of knee joint according to WORMS grading. Coronal plane **A** *MF* medial femoral condyle, *LF* lateral femoral condyle, *MT* Medial tibial plateau, *LT* Lateral tibial Plateau, *S* tibial spine area. Sagittal plane **B** *MFa* anterior medial femoral condyle, *MFc* central medial femoral condyle, *MFp* posterior medial femoral condyle, *MTa* anterior medial tibial plateau, *MTc* central medial tibial Plateau, *MTp* posterior medial tibial plateau. The subdivision on sagittal plane is similar of the lateral compartment

MRIs were assessed according to a pre-defined sheet which included the presence and location of bone bruises according to the whole-organ magnetic resonance imaging score (WORMS) for marrow abnormality [13]. Of the regions included in the original score, only those in sagittal plane were assessed for the purposes of this study, for a total of 13 areas. The femoral articular surface was divided into medial (MF) and lateral (LF) condyles, with the trochlear groove considered as part of MF. The boundary between MF and LF was defined by a plane aligned with the lateral wall of the femoral notch. MF and LF were each divided into three regions: anterior (a): extending from the anterior–superior osteochondral junction to the anterior margin of the anterior horn of the meniscus; central (c): extending from the anterior margin of the anterior horn of the meniscus to the posterior capsular attachment of the posterior horn of the meniscus; posterior (p): extending from the posterior capsular attachment of the posterior horn of the meniscus to the posterior-superior osteochondral junction. The tibial articular surface was divided into medial tibial plateau (MT) and lateral tibial plateau (LT), which were each divided into three equal regions: anterior (a), central (c) and posterior (p). The non-articulating portion of the tibial plateau beneath the tibial spines (S) was excluded from the assessment. The patella was considered as a single area (P) (Fig. 1). Bone bruises were defined as clearly defined areas of hyperintense signal, usually with a “flame” shape, in the subchondral bone in the T2-wheighted MRI sequence.

Based on the bone bruise location, the “Typical Pattern” was defined as a simultaneous bone bruise in the Central Lateral Femoral Condyle (LFc) and Posterior Lateral Tibial Plateau (LTp). Presence of only lateral tibial or lateral femoral or medial bone bruises were defined as “Atypical Patterns”.

### Statistical analysis

Statistical analysis was performed with MedCalc (MedCalc Software, Acacialaan, 22 Ostend, Belgium). Continuous variables were reported as mean ± standard deviation, while categorical variables were reported as absolute number and proportion of the total sample. Independent sample t-test was used to compare the continuous variable and Fisher exact test to compare dichotomous categorical variables. Due to the experimental and complex nature of the study design, the lack of previous studies with similar design and the strict inclusion criteria, an a-priori power analysis was not performed, and all the available patients were included.

The sample size was not large but reflects the total available findings during four consecutive seasons from one single country and therefore we could consider these results representative for the typical ACL injuries’ mechanisms in Qatar. Moreover, we used six analysts from different background with expertise in sport medicine for a better consistency and did consensus meetings for optimal accuracy.

## Results

### Patients’ characteristics

**Fig. 2:**
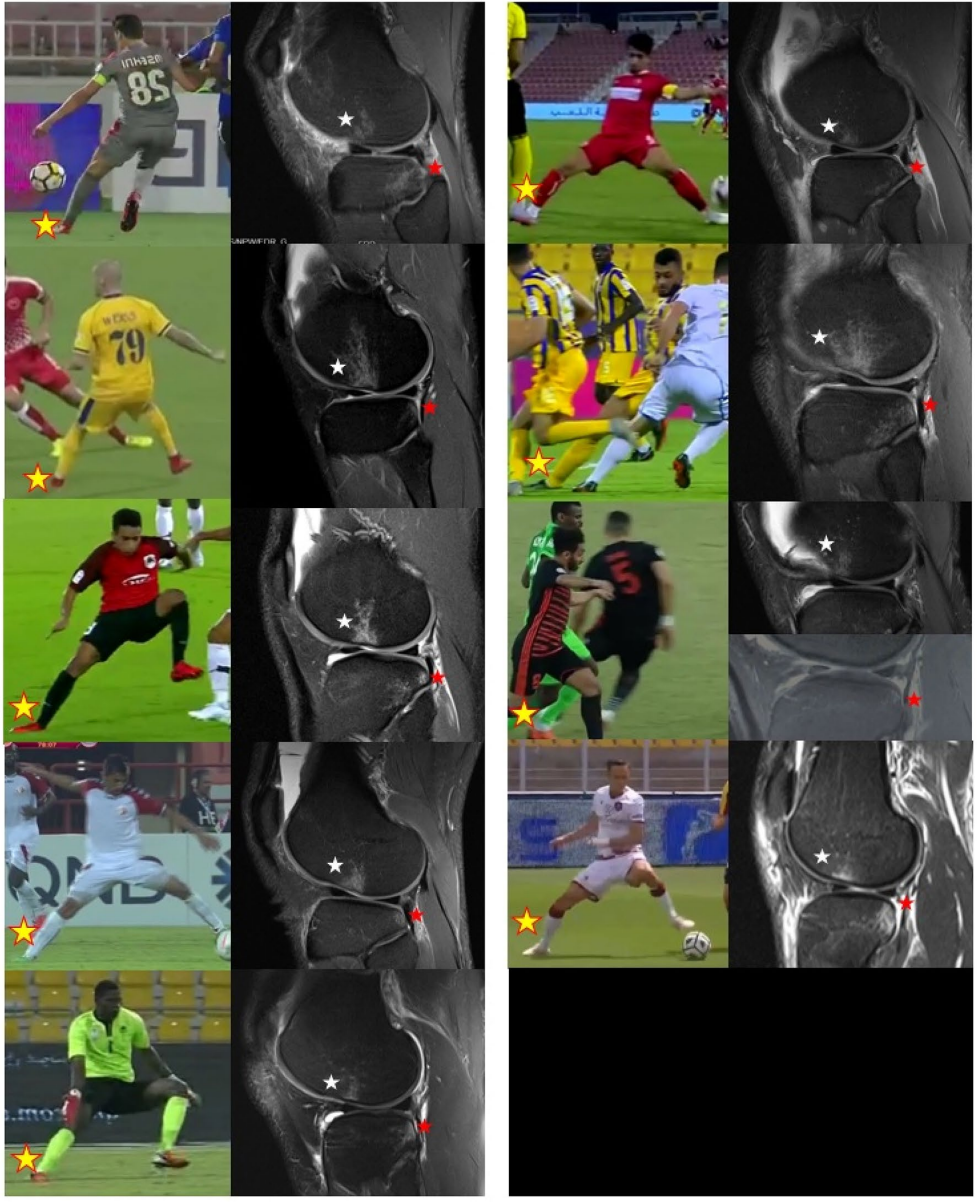
Correlation of injury mechanism and bone bruises of lateral compartment in the nine players with “pivoting” injury and “typical” bone bruise pattern; the yellow star indicates the injured leg, the white star indicates the central femoral bone bruise while the red star indicates the posterior tibial bone bruise. It is possible to note the consistent body and leg position of the injured leg at the time of injury, and the similar patterns of bone bruises

**Fig. 3:**
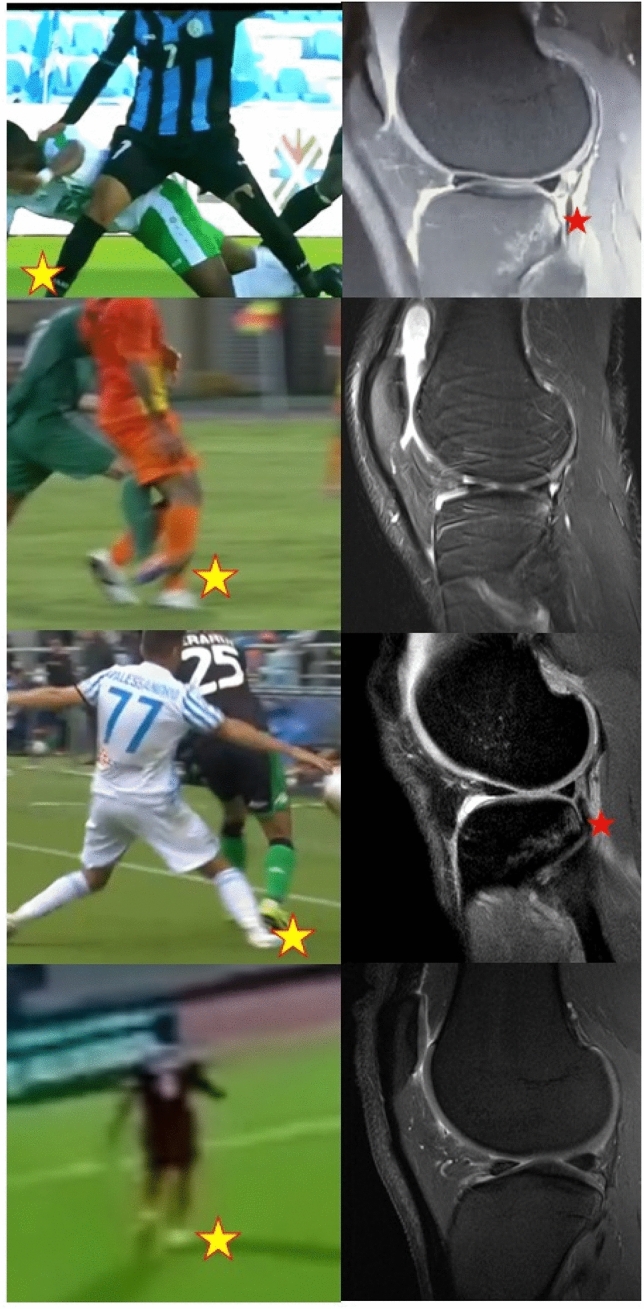
Correlation of injury mechanism and bone bruises of lateral compartment in the players with “contact” or “planting” injury mechanism and “atypical” bone bruise pattern; the yellow star indicates the injured leg while the red star indicates the posterior tibial bone bruise. In these cases, the body and leg position, the injury mechanism and the bone bruise patterns are heterogeneous

Overall, 15 footballers were included in the Qatar First League first, combined with 4 additional footballers from the Italian First League. Therefore, a total of 19 footballers (mean age 29.5 ± 3.6 years; 1 Goalkeeper, 5 Defenders, 9 Midfielders and 4 Forwards) were included (both MRI and ACL injury video). All patients had complete ACL rupture (100%). Nine athletes (47%) had a medial meniscus injury while 6 (32%) had lateral meniscus injury. Moreover, six (32%) had MCL injury ranging from Grade I to III while three (16%) had other concomitant injuries (two LCL sprain and one Patellar Tendon Rupture) Figs. 2 and 3.

### Video analysis

Overall, ACL injury mechanism was classified as “direct contact” in only 4 cases (21%) and “indirect” in the remaining 15 cases (non contact *n* = 9 and indirect contact *n* = 6) (79%).

The most common situational pattern was “pressing” (*n* = 7) accounting for the 47% of the “indirect” ACL injuries (Table 1). In terms of movement pattern, ten injuries (52%) occurred during a “Pivoting” movement (seven pressing, one dribbling, one tackled, one goalkeeping), whereas the remaining were classified as “Planting” in four cases, “Direct Blow” in four cases and “Landing” (Tables 2 and 3). Additional details, including biomechanics are reported in Tables 2 and 3.

**Table 1:** Summary of video analysis

Variables	Results (*n* = 19)
Injury mechanism	
Injury classification	Direct contact (*n* = 4)
	Indirect contact (*n* = 6)
	Non-contact (*n* = 9)
How many feet on the ground	One (*n* = 14)
	Two (*n* = 5)
Leg loading	Injured leg (*n* = 18)
	Uninjured leg (*n* = 1)
Horizontal velocity	Zero (*n* = 2)
	Low (*n* = 2)
	High (*n* = 15)
Vertical velocity	Zero (*n* = 17)
	Low (*n* = 1)
	High (*n* = 1)
Situational pattern	
Playing phase before injury	Offensive (*n* = 6)
	Defensive (*n* = 13)
Situational patterns	Pressing (*n* = 7)
	Tackling (*n* = 1)
	Tackled (*n* = 1)
	Recovery balance after kicking (*n* = 1)
	Landing from jump (*n* = 1)
	Others (*n* = 4)
	Direct contact (*n* = 4)
Movement pattern	Pivoting (*n* = 10)
	Planting (*n* = 4)
	Landing (*n* = 1)
	Direct Blow (*n* = 4)

**Table 2:** Details of video analysis

	Video	Injury mechanism	Playing phase	Situational pattern	Movement	Feet on the ground IF	Leg loading IF	Horizontal speed IF	Vertical speed IF	Tilt IC	Trunk rotation IC	Hip IC	Knee IC	Foot strike IC	Foot position IC	Tilt IF	Trunk rotation IF	Hip IF	Knee IF	Foot strike IF	Foot position IF
"Typical" bone bruise	Video 02	Indirect contact	Offensive	Tackled	Pivoting	1	Injured leg	2	0	15	Toward injured side	Abducted	Neutral	Flat	Extrarotated	15	Toward uninjured side	Abducted	Valgus	Flat	Extrarotated
	Video 03	Non-contact	Offensive	Pressing	Pivoting	1	Injured leg	2	0	15	Toward injured side	Abducted	Valgus	Flat	Extrarotated	10	Neutral	Abducted	Valgus	Flat	Extrarotated
	Video 04	Non-contact	Defensive	Other (missing the ball)	Planting	2	Injured leg	1	1	15	Neutral	Abducted	Neutral	Toe	Extrarotated	15	Neutral	Abducted	Valgus	Flat	Extrarotated
	Video 06	Indirect contact	Defensive	Pressing	Pivoting	1	Injured leg	2	0	10	Toward uninjured side	Abducted	Neutral	Flat	Extrarotated	5	Toward uninjured side	Abducted	Valgus	Flat	Extrarotated
	Video 07	Non-contact	Offensive	Other (dribbling)	Pivoting	1	Injured leg	2	0	5	Neutral	Abducted	Neutral	Flat	Extrarotated	0	Toward uninjured side	Abducted	Valgus	Flat	Extrarotated
	Video 09	Non-contact	Defensive	Other (goalkeeping)	Pivoting	1	Injured leg	2	0	10	Neutral	Abducted	Neutral	Neutral	Extrarotated	0	Toward uninjured side	Abducted	Valgus	Neutral	Extrarotated
	Video 12	Non-contact	Defensive	Pressing	Pivoting	1	Injured leg	2	0	0	Toward injured side	Abducted	Neutral	Flat	Extrarotated	0	Toward uninjured side	Abducted	Valgus	Flat	Extrarotated
	Video 13	Non-contact	Defensive	Pressing	Pivoting	1	Injured leg	2	0	-5	Toward injured side	Abducted	Neutral	Flat	Extrarotated	0	Toward uninjured side	Abducted	Valgus	Flat	Extrarotated
	Video 15	Non-contact	Defensive	Pressing	Pivoting	1	Injured leg	2	0	10	Toward uninjured side	Abducted	Neutral	Heel	Extrarotated	15	Toward uninjured side	Abducted	Valgus	Flat	Extrarotated
	Video 17	Indirect contact	Defensive	Landing from jump	Landing	1	Injured leg	0	2	\	\	\	\	toe	\	\	\	\	Valgus	Flat	\
	Video 18	Non-contact	Defensive	Pressing	Pivoting	1	Injured leg	2	0	-10	Toward injured side	Abducted	neutral	Flat	Extrarotated	-5	Toward uninjured side	Abducted	Valgus	Flat	Extrarotated
"Atypical" bone bruise	Video 01	Direct contact	Defensive	/	Direct blow	2	Both	0	0	\	\	\	\	\	\	\	\	\	\	\	\
	Video 05	Indirect contact	Defensive	Tackling	Planting	2	Injured leg	2	0	– 5	Neutral	Abducted	Neutral	Flat	Neutral	10	Toward uninjured side	Abducted	Valgus	Flat	Extrarotated
	Video 08	Direct contact	Defensive	/	Direct blow	2	Injured leg	2	0	\	\	\	\	\	\	\	\	\	\	\	\
	Video 10	Direct contact	Offensive	/	Direct blow	1	Injured leg	2	0	\	\	\	\	\	\	\	\	\	\	\	\
	Video 11	Indirect contact	Defensive	Other (missing the ball)	Planting	1	Injured leg	2	0	\	Neutral	Abduced	Neutral	Flat	Neutral	10	Neutral	Abducted	Valgus	Flat	Extrarotated
	Video 14	Direct contact	Defensive	/	Direct blow	1	Injured leg	1	0	\	\	\	\	\	\	\	\	\	\	\	\
	Video 16	Indirect contact	Offensive	Recovery balance after kicking	Planting	2	Injured leg	2	0	10	Neutral	Abducted	Valgus	Flat	Extrarotated	15	Toward uninjured side	Abducted	Valgus	Flat	Extrarotated
	Video 19	Non-contact	Offensive	Pressing	Pivoting	1	Injured leg	2	0	\	Neutral	Abducted	neutral	Flat	Extrarotated	\	Neutral	Abducted	Valgus	Flat	Extrarotated

**Table 3:** Details of Biomechanical Analysis

Video	Valgus collapse	Significant hip adduction relatively to IC	Trunk flex IC	Hip flex IC	Knee flex IC	Ankle flex IC	Trunk flex IF	Hip flex IF	Knee flex IF	Ankle flex IF
*Bone bruise pattern*										
"Typical"										
Video 02	No	Yes	5	60	50	\	5	65	65	\
Video 03	No	No	15	60	25	0	5	50	40	5
Video 04	No	Yes	-10	45	30	-15	0	55	60	5
Video 06	No	Yes	15	55	25	-30	10	55	40	10
Video 07	no	Yes	5	15	15	10	0	20	55	15
Video 09	No	Yes	10	50	15	10	10	55	30	15
Video 12	No	Yes	-15	55	10	-35	\	55	45	-10
Video 13	No	Yes	0	50	15	-35	-5	60	40	-5
Video 15	No	No	\	\	\	\	\	\	\	\
Video 17	\	\	-5	30	15	-20	-5	35	45	0
Video 18	Yes	Yes	5	40	5	-36	0	48	40	-3
"Atypical"										
Video 01	\	\	\	\	\	\	\	\	\	\
Video 05	No	Yes	− 5	50	15	\	0	50	35	-5
Video 08	\	\	\	\	\	\	\	\	\	\
Video 10	\	\	\	\	\	\	\	\	\	\
Video 11	No	Yes	NA	NA	NA	NA	NA	NA	NA	NA
Video 14	\	\	\	\	\	\	\	\	\	\
Video 16	Yes	Yes	NA	NA	NA	NA	NA	NA	NA	NA
Video 19	No	Yes	5	45	20	-10	3	45	40	3

### Bone bruises patterns

All MRIs were obtained within 7 days from trauma. The most commonly involved areas were the Posterior Lateral Tibial Plateau (LTp) in 16 cases (84%) and the Central Lateral Femoral Condyle (LFc) in 11 cases (58%). Three patients (16%) had bone bruise in the Posterior Medial Tibial Plateau (MTp) while none (0%) had bone bruise in the Medial Femoral Condyle. Based on the bone bruise pattern, 11 (58%) had simultaneous LFc and LTp and were defined “Typical” while 8 (42%) had other locations or no bone bruise and were defined “Atypical” (Table 4).

**Table 4:** Patterns of Bone Bruises

Lateral femoral condyle	
Anterior (LFa)	1 (5%)
Central (LFc)	11 (58%)
Posterior (LFp)	0 (0%)
None	7 (37%)
Lateral tibial plateau	
Anterior (LTa)	1 (5%)
Central (LTc)	1 (5%)
Posterior (LTp)	16 (84%)
None	2 (11%)
Medial femoral condyle	
Anterior (MFa)	0 (0%)
Central (MFc)	0 (0%)
Posterior	0 (0%)
None	0 (0%)
Medial tibial plateau	
Anterior (MTa)	0 (0%)
Central (MTc)	0 (0%)
Posterior (MTp)	3 (16%)
None	16 (84%)
Patella (P)	
Medial or lateral facet (P)	1 (5%)
None	18 (95%)
Bone bruise patterns	
"Typical" pattern	11 (58%)
LFc + LTp	9 (47%)
LFc + LTp + MTp	2 (11%)
"Atypical" pattern	8 (42%)
LTp	4 (21%)
LTp + MTp	1 (5%)
LTa + LTc + LFa + LFc + P	1 (5%)
None	2 (11%)

When the injury mechanisms were stratified based on the bone bruises patterns, patients with the “Typical” pattern had an “indirect” ACL mechanism in all cases (100%) compared to four cases (50%) in athletes with “Atypical” patterns (*p* = 0.0181). Moreover, 9 out of 11 injuries (82%) of athletes with “Typical” pattern occurred with a “Pivoting” action”, in contrast to only 1 case (12%) in those with “Atypical” bone bruise pattern (*p* = 0.0055) (Table 5).

**Table 5:** Analysis based on Bone Bruises Patterns

	Typical bone bruise pattern (*n* = 11)	Atypical bone bruise pattern (*n* = 8)	*p*-value
Age	29.8 ± 2.9	29.0 ± 4.5	n.s
Role			n.s
Goalkeeper	0 (0%)	1 (12%)	
Defender	3 (27%)	2 (25%)	
Midfielder	6 (55%)	3 (38%)	
Forward	2 (18%)	2 (25%)	
Medial Meniscus Lesion			n.s
No	6 (55%)	4 (50%)	
Yes	5 (45%)	4 (50%)	
Type	4 PH, 1 Ramp	2 PH, 2 Ramp	
Lateral Meniscus Lesion			
No	6 (55%)	7 (88%)	n.s
Yes	5 (45%)	1 (12%)	
Type	2 AH, 2 Body, 1 PR	1 PR	
MCL Injury			n.s
No	7 (64%)	6 (75%)	
Yes	4 (36%)	2 (25%)	
Type	2 Gr I, 1 Gr II, 1 Gr III	1 Gr II, 1 Gr III	
Cartilage Injury			n.s
No	11 (100%)	6 (75%)	
Yes	0 (0%)	2 (25%)	
Type	None	1 MFC, 1 LFC	
Other Injuries			n.s
No	9 (82%)	7 (88%)	
Yes	2 (18%)	1 (12%)	
Type	1 LCL srain, 1 PT rupture	1 LCL sprain	
Injury Type			= 0.0181*
Direct	0 (0%)	4 (50%)	
Indirect	11 (100%)	4 (50%)	
Injury Mechanism			
Pivoting	9 (82%)	1 (12%)	= 0.0055*
Other	2 (18%)	7 (88%)	
Direct blow	0	4	
Planting	1	3	
Landing	1	0	

## Discussion

The most relevant finding of the present study is the characterization of a well-defined tibiofemoral BB pattern in indirect ACL injuries during footballers' pivoting with sudden change of direction/deceleration, through MRI and video-analysis correlation. To our knowledge, this is the first study to correlate specific ACL injury mechanisms with MRI findings in professional footballers.

The first description of BB patterns by Sanders et al. [21] revealed their role as footprints of ACL injury mechanisms. Since then, several studies have shown how valuable they are for the ACL surgeon and their almost ubiquitous presence in these injuries [2, 16, 19, 20, 26]. Biomechanically, the forces participating in the ACL injury are knee valgus, anterior tibial translation, lateral tibial translation, and internal tibial rotation [1, 24, 27]. Several investigators have reported the highest prevalence, severity, and volume of BB in the lateral knee compartment, attesting to the significant contribution of valgus forces in the ACL injury [17, 20, 21, 23, 24]. Similarly, "kissing lesions" in the central area of the lateral femoral condyle and posterior area of the lateral condyle demonstrate the tibia's anterior translation and internal rotation during indirect injuries [23].

In line with previous reports [22, 23, 25, 27], our results show that combined central lateral femoral condyle and posterior lateral tibial plateau BB pattern to be the result of indirect ACL injury mechanism in 100% of cases, and was the primary or typical BB pattern accounting for 58% of the included footballers. Identifying this initial pattern in patients undergoing ACL reconstruction surgery may have a potential role in guiding footballers' rehabilitation, focusing on valgus knee collapse prevention exercises and improving cutting task biomechanics, and recognizing individuals at higher risk of reinjury.

On the other hand, according to recent studies, the presence of three or four contusions and the involvement of medial compartment BB are more prevalent than previously thought [16]. The involvement of the medial compartment in BB patterns has been associated with a varus compensatory alignment and countercoup injury, when the femur and tibia reduce and both bones contact after the ACL rupture [15]. Time to MRI assessment and MRI quality have been proposed as reasons for the underreporting of these injuries in former studies, as the medial compartment BB, secondary with less severity and volume, could resolve earlier or be missed [10, 16, 24].

Kim-Wang et al., in a cross-sectional study comprising 136 patients undergoing ACL reconstruction, found medial compartment BB in 72% of patients in his series and the presence of 3 or 4 contusions in 65% of them, with a prevalence of 35% involving all compartments [16]. Similarly, Qiu et al., in a retrospective analysis of 93 patients with an ACL injury, reported medial tibial plateau involvement in 69.8% and medial femoral condyle BB in 49.4% of patients [23]. Although medial compartment-only patterns represented only 5.3% of patients, four contusion patterns were the most common, accounting for 36.5% of patients. Last, in Lattermann et al. study, they found three or four contusions in 49% of their cases and 11% comprising all compartments [18].

In contrast, medial compartment BB involvement in our series was observed only in three patients (15.7%) and exclusively on the posterior medial tibial surface. Two of them were associated with the central lateral femoral condyle and the posterior medial tibial plateau and considered typical BB patterns. No medial femoral condyle BB was present in our series, and only three (15.7%) had three or four contusion patterns. Video-analysis could explain the limited number of medial compartment involvement, since only 4% of ACL injuries in male professional football players showed a knee varus alignment at injury frame [5], and most of injuries occurred with similar and consistent rotatory patterns, rather than with coronal deformities.

Other BB patterns found in this study were heterogeneous. However, they were characterized by the lack of lateral femoral BB involvement or no BB at all. These atypical patterns might be the consequence of direct or different indirect injuries mechanisms, such as hyperextension, lower valgus forces, or lower injury-generating forces in general.

As for our hypothesis, different ACL injuries patterns correlated with distinct MRI and BB patterns. The classic indirect ACL injury (non-contact or indirect contact) happening during a “pivoting” with a change of direction/deceleration is associated in all the cases but one (nine out of ten; 90% of cases) to a double bone bruise pattern on lateral compartment, therefore indicating a specific loading pattern. This is of further interest as this specific inter-segmental body relationship at the time of injury [5] may be correlated to functional outcomes following ACLR. It has been recently demonstrated on a cohort of 118 professional football players, that a non-contact ACL injury mechanism was linked to a sevenfold increase in the likelihood of a 2nd ACL injury [7]. Of further interest, an isolated ACL injury, in conjunction with a non-contact mechanism was an additional risk factor for a second knee hit, bringing the 2nd ACL injury rate to 42% [7]. This study clearly demonstrates that specific videos patterns of ACL injury correlate with a different joint loading and therefore it is possible that an indirect injury with “Typical” double bone bruise may predispose the athlete to an increased risk of recurrences after treatment.

What can the video-analysis tell us about atypical patterns or direct injuries? Our data suggest that the whole movement pattern, and not the single joint kinematic should be considered. When considering the “Atypical” BB presentation in indirect injuries three out of four were the results of “Planting” defined as foot planting to the ground without any clear change of direction and high horizontal velocity, typical features of the “Pivoting” movement patterns. An important feature of the pivoting pattern is indeed the trunk rotation towards the uninjured side at injury frame, which is coupled with medial thigh motion (femoral internal rotation and adduction) and tibial abduction on an externally rotated foot. It is likely that this unique combination of kinematic factors, with an internal rotation of the upper body, is linked to what happen within the knee joint and resulting with a distinct BB pattern.

These new findings raise questions whether or not ACL injured athletes with “Typical” BB patterns should be treated differently after surgical stabilization. This sub-group of patients may need a high volume of neuromuscular training and increased attention on movement patterns, particularly related to the promotion to deceleration tasks or cutting mechanics [6, 9], which should be implemented in the mid and late-stage rehabilitation process [4].

The question remains then what video-analysis can tell us about injury mechanisms and their correlation with associated injuries? One of the present study's major strengths is the timing of MRI assessment, performed within seven days from the injury. This represents an almost unique feature, since most of the studies evaluating bone bruises usually included MRIs performed within weeks or even months since injury. This is relevant since a longer time from injury to MRI could underestimate the presence of bone bruises. Moreover, the use of injury video-analysis through video footages allowed a correlation between MRI features and the actual injury mechanism, which is supposed to be more reliable than injury description by patient recall. However, the most important limitation is represented by the small sample size, which did not allow for a sophisticated statistical analysis, especially regarding concomitant injuries such as meniscal, cartilage and collateral ligaments; these events were not common and a proper analysis was not possible. Finally, the bi-dimensional nature of the video did not allowed a perfect tridimensional assessment of knee position at the time of injury, differently from model-based image-matching analysis, which could have allowed the precise estimation of tibial translation and rotation. However, it was not the aim of this study to analyze the ACL injuries in depth, but rather to highlight the correlation between the mechanism of ACL injury with MRI Bone Bruise features in elite football.

The results of this study could assist the clinician in defining individualized treatment and prevention strategies for professional athletes with an ACL knee injury (based upon the available MRI imaging and video footage).

## Conclusions

A well-defined and consistent bone bruise pattern involving the posterior tibial plateau and central femoral condyle of lateral compartment is present in footballers that sustained non-contact and indirect ACL injuries during pivoting with sudden change of direction/deceleration, while heterogeneous patterns were present in those with direct contact or injury mechanisms involving high horizontal velocity.
